# Engineering Genetically Encoded FRET Sensors

**DOI:** 10.3390/s140711691

**Published:** 2014-07-02

**Authors:** Laurens Lindenburg, Maarten Merkx

**Affiliations:** Laboratory of Chemical Biology and Institute of Complex Molecular Systems (ICMS), Department of Biomedical Engineering, Eindhoven University of Technology, Eindhoven 5600MB, The Netherlands; E-Mail: laurens.lindenburg@googlemail.com

**Keywords:** FRET, sensors, directed evolution, protein engineering, multiparameter imaging, fluorescent proteins

## Abstract

Förster Resonance Energy Transfer (FRET) between two fluorescent proteins can be exploited to create fully genetically encoded and thus subcellularly targetable sensors. FRET sensors report changes in energy transfer between a donor and an acceptor fluorescent protein that occur when an attached sensor domain undergoes a change in conformation in response to ligand binding. The design of sensitive FRET sensors remains challenging as there are few generally applicable design rules and each sensor must be optimized anew. In this review we discuss various strategies that address this shortcoming, including rational design approaches that exploit self-associating fluorescent domains and the directed evolution of FRET sensors using high-throughput screening.

## 1. Genetically Encoded Fluorescent Sensors

Genetically encoded intracellular fluorescent sensors have been developed to image a range of intracellular parameters including the concentration of many chemical species [1–3]. As these sensors are protein-based, they can be easily targeted to subcellular locations by appending a localization tag to them and they are less invasive than other classical single cell interrogation techniques such as microinjection and patch clamping, as their expression simply requires transfection of their encoding DNA. These sensors all employ the large pallet of fluorescent proteins (FPs) developed since the discovery of Green Fluorescent Protein (GFP). The development and diversity of fluorescent proteins themselves are beyond the scope of this Review, and the reader is hence referred to several excellent recent reviews on these topics [4–6]. Genetically encoded fluorescent sensors can be divided into two categories: those that modulate the fluorescence of a single FP and those that display changes in FRET between a donor and acceptor FP.

**Figure 1.**
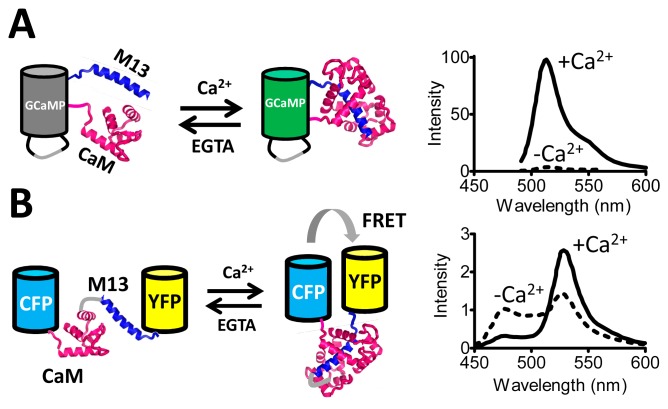
Comparison of a single FP-based (A) and a FRET-based (B) genetically-encoded Ca^2+^ sensor. (**A**) GCamP consists of a circularly permutated GFP in which calmodulin (CaM) and M13 are grafted into the beta-barrel using the newly available N- and C-termini. In absence of Ca^2+^, the FP is only dimly fluorescent, due to solvent quenching of the fluorophore. Ca^2+^ binding and the concomitantly induced M13-calmodulin interaction excludes solvent from the chromophore, dramatically increasing fluorescence intensity. The graph on the right shows the typical change in emission spectrum of GCaMP and related sensors upon Ca^2+^ addition. (**B**) The Cameleon FRET sensor for Ca^2+^ also uses CaM and M13, but now sandwiched between CFP and YFP. The Ca^2+^-binding-induced intramolecular CaM-M13 interaction results in an increase in FRET. The graph on the right shows the typical emission spectra in presence and absence of Ca^2+^ seen with Cameleon and related sensors.

The single FP sensors rely on modulation of the degree of exposure of the FP chromophore to the solvent, which has a large effect on its fluorescent properties. Some of the first sensors developed along this principle were sensitive to pH [7] and to redox state [8]. With the development of circular permutants of GFP it became possible to graft protein domains onto the β-barrel [9] such that these domains' conformation directly modulates the chromophore's access to solvent. For example, calmodulin and M13, which undergo a Ca^2+^-dependent interaction, were fused to the new N- and C-termini of circularly permutated GFP, generating the GCaMP probe [10], whose fluorescence is quenched in the absence of Ca^2+^ (Figure 1A). GCaMP has spawned a series of ever improving Ca^2+^ probes [11–13]. Other useful single FP sensors developed since include the ATP to ADP ratio sensor Percival [14,15], the dual pH and Cl^−^ sensor ClopHensorN [16], the membrane potential sensor ArcLight [17,18], the H_2_O_2_ sensor HyPer [19], and the glutamate sensor iGluSnFR [20]. Most single FP-based sensors are intensiometric which makes them less robust as fluctuations in sensor concentration may be mistaken for actual signal change. However, a few single FP-based sensors have been developed that are excitation-ratiometric, changing their relative excitation at two different wavelengths as a function of ligand concentration [21,22]. In the single FP sensors, the necessity for allosteric coupling of ligand binding to modulation of the chromophore environment introduces a serious protein engineering challenge. Nevertheless, several single FP-based sensors have been developed that showed excellent signal to noise ratios [13,20].

Genetically encoded Förster Resonance Energy Transfer (FRET) sensors, the subject of this review, consist of two fluorescent domains flanking a recognition domain and forming a single polypeptide chain. Ligand binding to the recognition domain changes its conformation, resulting in a change in the relative orientation of the FP-FP geometry, and thus a change in FRET. FRET is defined as radiationless energy transfer between two chromophores as a result of long-range dipole-dipole interactions [23]. The FRET efficiency depends not only on the distance and the relative orientation (κ^2^) of those two dipoles, but also on their intrinsic spectral properties. These factors are the overlap (J) between the donor's emission and the acceptor's absorption spectra, normalized to the acceptor's extinction coefficient and the quantum yield of the donor. The refractive index (n), which is the ratio of the speed of light in vacuum relative to its speed in the medium through which the radiationless energy transfer takes place, also affects the FRET efficiency (E). The intrinsic fluorescent properties of the donor and the molar absorptivity of the acceptor can be used to calculate the distance between the dipoles at which energy transfer is 0.5 (*i.e.*, where the donor's emission intensity is reduced to 50% of its value in absence of acceptor), a convenient measure of a FRET pair's suitability, and known as R_0_ (Equation (1)):

(1) $$R_{0}=0.211\cdot {\left[n^{-4}\cdot \kappa^{2}\cdot \phi \cdot J\right]}^{\frac{1}{6}}$$

A value of 1.4–1.5 is often used for the refractive index n, as this value is intermediate between the value of 1.6 determined for the protein interior and the 1.33 value for the aqueous environment [24,25]). κ^2^ is typically assumed to be 2/3, a value representing freely rotating dipoles. The actual efficiency of energy transfer (*E*) will depend on the distance (*r*) between the fluorophores, as given by Equation (2):

(2) $$E=\frac{{R_{0}}^{6}}{{R_{0}}^{6}+r^{6}}$$

Typical values for FP pairs' R_0_ range between 40 and 60 Å [26,27]. For fluorescent proteins, a lower limit in *r* of about 30 Å is set by the width of the beta-barrel protein shell surrounding the fluorophore. While the acceptor's fluorescent quantum yield thus has no influence on the efficiency of energy transfer itself, a high acceptor quantum yield is beneficial for the robust detection of FRET with the commonly used ratiometric method, where changes in donor to acceptor intensities are monitored [28]. Also important for detection is a low degree of bleed through of the donor and the acceptor emission into each other's detection channels [29]. Several FRET-based sensors have been used that employ chromoproteins such as the yellow REACh [30] with negligible fluorescence as acceptors [31,32]. A disadvantage of this approach is that it is intensiometric, requiring imaging of the donor life time to accurately monitor changes in FRET. An advantage of these probes is that they use a smaller part of the available spectral range, making them particularly suitable for multiparameter imaging [32].

A FRET sensor for Ca^2+^, Cameleon, was one of the first sensors to be constructed using the principle of FRET between two fluorescent domains (Figure 1B). This construct is a single polypeptide chain consisting of ECFP, calmodulin (CaM), M13 and EYFP. Upon binding of Ca^2+^ to CaM, M13's affinity for CaM is increased, resulting in the wrapping of the M13 around CaM and changing the relative geometry of the FPs, leading to an increase in FRET [33]. As Cameleon's output signal was emission ratiometric, measurements were not sensitive to fluctuations in sensor concentration, optical path length or excitation intensity [33].The dynamic range (DR) (ΔR/R_min_) of this sensor was a very reasonable 70%. However, achieving this DR required extensive testing of different designs. Notably, the composition of the short peptides linking each of the protein domains turned out to be crucial for sensor functioning, with many deletions, insertions and substitutions tested at these positions [33].

Many of the FRET sensors described since this prototype have required a similar effort, highlighting the fact that despite the seemingly modular design, FRET sensor development still involves trial-and-error-based, stepwise improvements. The goal in FRET sensor development is to make the *change* in distance *r*: (i) as large as possible and (ii) centered around R_0_ as closely as possible. An additional means of modulating the efficiency of energy transfer is achieved through a change in R_0_ resulting from a change in dipole-dipole orientation.

Note that *semi-synthetic* FRET sensors, that is constructs employing a natural protein domain coupled to small molecule dyes, have also been developed. Indeed, FlCRhR, a FRET sensor for cAMP, predated Cameleon by several years [34]. More recently, technologies such as FlAsH [35] and SNAP-tag [36] have made site-specific incorporation of synthetic dyes in natural protein domains straightforward. These strategies not only allow novel sensor approaches to be developed [37,38] but also allow sensitive monitoring of changes in protein conformation [39].

These approaches are beyond the scope of this review, which focuses on the entirely genetically encoded sensors. The high number of genetically encoded FRET sensors developed to date cannot be summarized in this short review; instead, the emphasis is on strategies for the efficient *development* of FRET sensors. The next section will deal with strategies undertaken to increase the ligand-dependent change in energy transfer in FRET sensors.

## 2. Design of FRET Sensors Based on Single Recognition Domains

**Figure 2.**
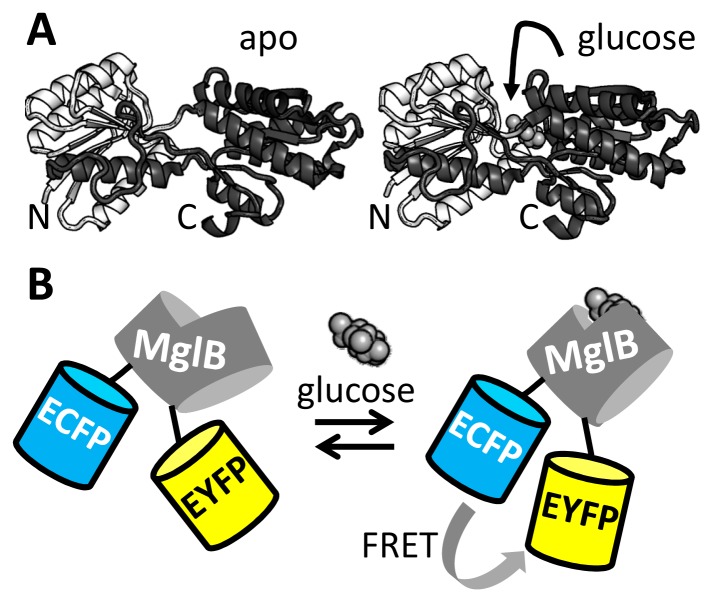
A genetically encoded FRET sensor for glucose based on the periplasmic binding protein MglB. (**A**) Crystal structure of apo-MglB (PDB 2FW0) and glucose-bound MglB (PDB 2FVY) [51]. The N-terminal lobe is colored in white while the C-terminal lobe is colored dark gray. Note the lack of any large difference in orientation or distance of the N and C-termini between the apo and glucose-bound states. (**B**) Schematic representation of the mechanism of action of a glucose FRET sensor based on MglB. Although in this figure a FRET increase is depicted upon glucose binding, a decrease in FRET was also observed in some other variants [45].

Following the construction of the first FRET sensor for Ca^2+^ [33], genetically encoded FRET sensors have been developed for a wide range of ions [1,40], small molecules [2], enzyme activities [41,42] and membrane potential [43]. These probes typically employ protein domains that are known to change conformation upon ligand binding, with FPs fused to either end of this domain, in the hope that the change of conformation in the recognition domain is transduced to a change in FP relative distance and/or orientation. However, initial designs typically yield sensors with poor DR, necessitating laborious and time-consuming rounds of trial-and-error based improvement. For example, a number of FRET sensors have been constructed using the bacterial periplasmic binding proteins (PBPs) as sensing module. Despite possessing seemingly ideal properties such as solubility, a well-understood venus fly-trap-like motion of two lobes upon ligand binding and a large selection of binding specificities for many small molecules of interest [44], PBPs often make poor FRET sensor recognition domains in simple fusion constructs. As the N and C-termini of the PBP are often located on the same lobe, they show little change in their relative orientation and distance upon ligand binding (Figure 2A).

In a series of papers [45–49], Frommer and colleagues introduced generally applicable strategies to improve the poor performance of such fusion constructs for a wide selection of small molecule ligands. For example, the DR of a FRET sensor based on MglB [46], a glucose binding PBP, was initially just 10% (Figure 2B). The sensor was improved by truncating the linkers between MglB and the fluorescent domains, yielding a glucose sensor with a 20% DR [45]. Also, improvements were noted when the ECFP donor fluorescent domain was inserted at rationally selected sites within the MglB domain [45]. The improvements were ascribed to a tighter allosteric coupling of the ligand-induced conformational change of MglB to the orientation of the FP domains. In particular, the linker truncation and recognition domain insertion should reduce the fluorescent domains' degrees of freedom for rotation. A further improvement in sensor DR (10 times better than the original sensor) was achieved by combining both strategies of linker truncation and domain insertion into a single sensor, FLII^12^Pglu-700μδ6 [47]. Another “trick” that can be used to improve the response of PBP-based sensors is circular permutation of the recognition domain with the aim of having the new PBP N and C-termini reside on different lobes of the PBP. It was shown that FRET sensors using six different PBPs could be improved in this way, including two that were non-functional in absence of the PBP circular permutation [50].

**Figure 3.**
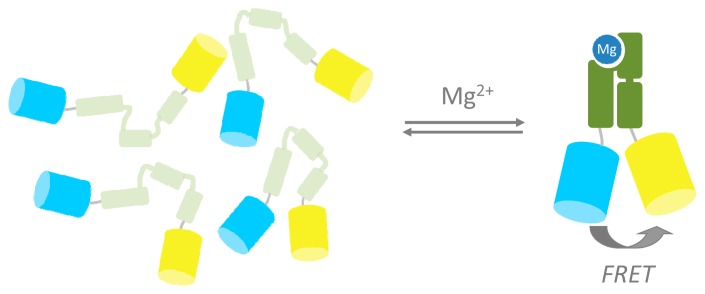
Intrinsically unfolded protein as input domain for FRET sensors. In the absence of Mg^2+^ the molten-globule state of the N-terminal part of HsCen3 results in little FRET. Binding of Mg^2+^ induces formation of a well-defined EF-hand fold, which decreases the distance between Cerulean and Citrine and thus increases FRET. Figure adapted from [55].

A promising strategy in FRET sensor design is the use of ligand-induced folding of sensing domains. The Palmer and Eide groups demonstrated this by sandwiching a Zn^2+^ finger domain in between ECFP and EYFP. A large increase in FRET was observed upon addition of Zn^2+^ as this metal induced folding of the initially unstructured Zn^2+^ finger [52,53]. Kohn and Plaxco have shown that conformationally silent ligand binding domains can be systematically destabilized by deletion of C-terminal residues such that the domain only folds in the presence of its ligand, providing a generic mechanism to generate ligand binding domains for FRET sensor proteins [54]. Recently, our group reported a genetically encoded FRET sensor for intracellular Mg^2+^ (MagFRET) based on a truncated version of the human centrin protein HcCen3. The N-terminal part of this protein forms a molten globule in its apo state, but folds into a compact, EF-hand-like fold upon binding Mg^2+^ (Figure 3). Fusion of Cerulean and Citrine fluorescent domains to either end of the N-terminal domain of HsCen3 in MagFRET thus yielded a sensor that undergoes an increase in FRET upon binding Mg^2+^, corresponding to a 50% increase in emission ratio [55]. Mutations at the HsCen3 metal binding sites allowed tuning of the sensor's *K*_d_ from 150 μM to 15 mM Mg^2+^. The sensor was expressed in the cytosol of mammalian cells but manipulation of the high free Mg^2+^ concentration (thought to be 0.2–1.5 mM) in intact cells proved to be difficult. Although the large transition in conformation simplifies FRET sensor design, a disadvantage of this approach is the sensitivity of the molten globule to ionic strength, affecting the emission ratio in the sensor's apo-form.

## 3. FRET Sensors Based on Ligand-Induced Domain-Domain Interactions

With the exception of sensors based on ligand-induced folding, most single recognition domain-based FRET probes rely on the subtle internal rearrangements of an individual domain's tertiary structure to change the relative distance and orientation between two FPs. Sensors that are based on multiple domains that undergo ligand-dependent intramolecular protein-protein interactions often yield better results as these sensors exploit the movement of large globular domains relative to one another.

One of the early examples of such a sensor is provided by the first genetically encoded FRET sensor for Ca^2+^, Cameleon, mentioned above (Figure 1B). Miyawaki *et al.* had found that a combination of deletions, insertions and substitutions at linker sites between CaM-M13 and the FPs was crucial to achieve sufficient DR. Their best effort at that time had a DR of 70% [33]. In the years that followed, improved Ca^2+^ FRET sensors were developed. Replacement of the photolabile and pH sensitive EYFP with Citrine [56] or Venus [57] improved the sensors' *in situ* performance. Replacement of the CaM-M13 sensing module by the protein Troponin C from chicken helped to improve DR and served to avoid undesirable interactions that might otherwise occur with a FRET sensor employing a human-derived recognition domain [58]. Also of importance were mutations, both in Troponin C [59] and CaM [60], that increased the specificity for Ca^2+^ over Mg^2+^. A significant ∼4-fold improvement in the DR of these Ca^2+^ sensors was achieved by replacing the acceptor with a circular permuted acceptor (cpAcceptor), either cpVenus [61] or cpCitrine [59]. Exactly how the cpAcceptor strategy improved the sensors remained unclear. It has been argued that introduction of a cpAcceptor results in a greater difference in orientation of the FP pair between the Ca^2+^-loaded and the apo state, modulating R_0_ itself [61]. However, an alternative hypothesis is that with a cpAcceptor, a hydrophobic face of this protein becomes available for complex formation with the donor FP, resulting in a higher level of FRET in the Ca^2+^-loaded state of the sensor [62].

**Figure 4.**
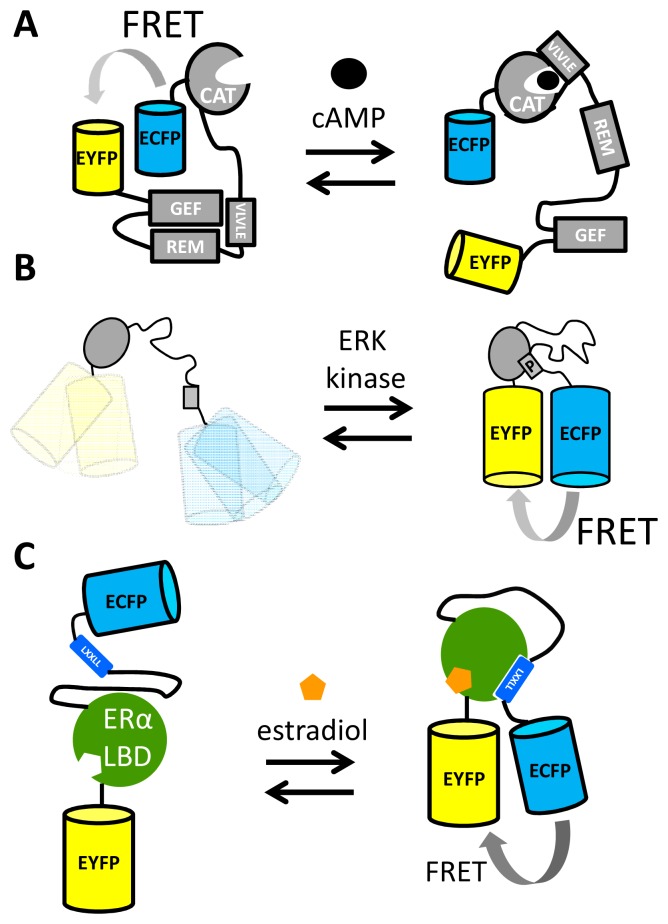
Ligand-dependent protein-protein interactions of recognition domains often yield good FRET sensors. (**A**) A FRET sensor for cAMP exploiting the multidomain Epac1 cAMP-binding protein. The large conformational rearrangement of Epac1 upon binding cAMP results in a large change in FRET between fluorescent proteins fused to the termini of Epac1 [65]. (**B**) In kinase sensors, interaction between a phosphobinding domain and a phosphorylated substrate peptide typically results in an increase in FRET [74]. (**C**) A FRET sensor for agonists of the estrogen receptor uses the ligand binding domain of ERα, together with a co-activator peptide, to bring about a large increase in FRET between ECFP and EYFP upon an estradiol-binding induced co-activator-LBD interaction [76].

Another important second messenger for which FRET sensors have been developed is cAMP, which is synthesized from ATP upon extracellular stimulation of G-protein-coupled receptors (GPCRs) [63]. While early efforts in the field focused on using the cAMP-dependent protein kinase (PKA) as a recognition domain in sensors, this protein had the disadvantage of possessing multiple cAMP binding sites and an affinity that was too high [34,64]. Jalink and coworkers realized that the “Exchange protein directly activated by cAMP” (Epac1) had the fortuitous property of a large change in conformation upon cAMP binding [65]. Simply fusing ECPF and EYFP to either end of Epac1 yielded a functional sensor, which was further improved by deleting a membrane anchoring sequence and introducing mutations that rendered Epac catalytically inactive (Figure 4A). This sensor, named CFP–Epac(δDEP-CD)–YFP, had a DR of 45% and an *in vitro K*_d_ for cAMP of 14 μM [65], two orders of magnitude higher than that of an earlier effort [34]. Upon binding of cAMP to an N-terminal catalytic domain (CAT) of the sensor, a protein-protein interaction between CAT and a VLVLE motif resulted in an extended conformation of the protein, as the regulatory domain (REM) and the guanine nucleotide exchange factor (GEF) domain no longer interacted [65]. The importance of the intramolecular, interdomain interactions seen in the CFP–Epac(δDEP-CD)–YFP mechanism of action is illustrated by the fact that a cAMP sensor that employed only the catalytic domain, Epac2-camps, displayed a significantly reduced DR [66]. Further attempts to improve the cAMP sensor included the use of a double acceptor [67] (cpVenus and Venus) and introduction of the brighter donor mTurquoise [68], finally resulting in ^T^Epac^VV^, a bright sensor with a DR of 100% [69].

FRET sensors for another critical component in cell signaling, kinase activity, have been reported for a number of kinases such as PKA [70], tyrosine kinases such as the insulin receptor [71], Src [72], extracellular signal-regulated kinase (ERK) [73–75] and protein kinase C (PKC) [42]. All these reporters of kinase activity share a common mechanism of action: a kinase “substrate” peptide that becomes phosphorylated and then is recognized by and becomes bound to a binding domain (Figure 4B).

An important factor that affects the DR of FRET sensors for kinase activity is the basal (*i.e.*, prestimulation) level of phosphorylated sensors. Recently, a strategy was introduced to minimize this level. Komatsu *et al.* reasoned that by increasing the length of the linker connecting the phosphobinding domain with the substrate peptide, the reduced effective concentration would encourage faster dissociation [74]. Faster dissociation of the phosphobinding domain from the phosphorylated peptide would allow phosphatases to act on the peptide and return the sensor to the non-phosphorylated off-state. An added benefit of the longer linker was a reduced level of FRET in the off state. Impressively, the authors demonstrated the benefit of their approach by improving the DR of sensors for the activities of four different kinases, as well as introducing several novel functional kinase sensors [74]. For example, EKAR, a FRET sensor for ERK activity [73], was improved 4-fold [74].

Nuclear Receptors (NRs) are transcription factors whose activity can be modulated by small molecule ligands [77]. A large number of NRs are known, with each NR binding specific signaling molecules such as hormones, intracellularly. Upon binding ligand, the NR ligand binding domain (LBD) increases its affinity for co-activator proteins, binding these co-activators via their canonical LXXLL motif. This ligand-dependent protein-protein interaction was exploited in FRET sensors consisting of ECFP, LBD, an LXXLL containing peptide and EYFP (Figure 4C). For example, a FRET sensor responsive to 17β-estradiol (E2) and other Estrogen Receptor (ER) agonists was constructed using the LBD from ERα. The sensor displayed a 40% DR, binding E2 with an EC_50_ of 80 nM [76]. Similar FRET sensors based on the LBDs of the androgen receptor [78] and the glucocorticoid receptor [79] have also been successfully constructed.

## 4. FRET Sensors Based on Interacting Fluorescent Domains

**Figure 5.**
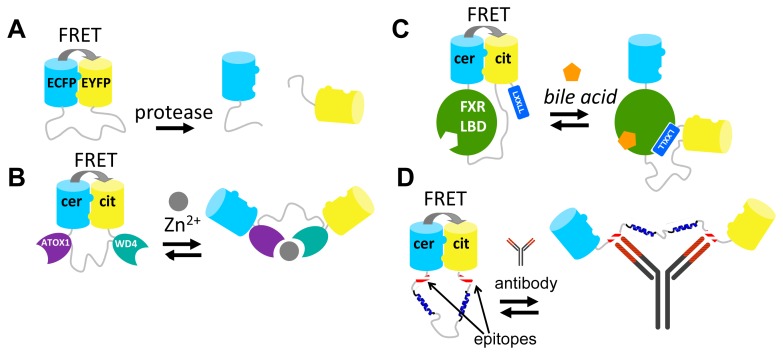
FRET sensors based on self-associating fluorescent domains. These FRET sensors employ the S208F/V224L (A,B) or Q204F/V224L (C,D) hydrophobic mutations on the ECFP-EYFP (A) or Cerulean (cer)-Citrine (cit) (B-D) FRET pairs, ensuring an initial self-association and thus a high starting level of FRET. (**A**) Protease sensor, cleavage of the intervening linker sequence disrupts the fluorescent domain complex [81]. (**B**) eCALWY Zn^2+^ sensor, formation of an ATOX1-WD4-Zn^2+^ complex disrupts the interaction between the fluorescent domains [84]. (**C**) Bile acid sensor, bile acid-dependent binding of LXXLL to FXR-LBD results in disruption of the fluorescent domain complex [85]. (**D**) Antibody sensor, binding of antibody to the epitopes adjacent to Cerulean and Citrine disrupts the fluorescent domain complex [86]. Picture adapted from [80].

Although the strategies described above have provided many useful and robust FRET sensors, their development required a lot of time and effort. Moreover, most of these approaches lack modularity and are therefore not generally applicable. For example, despite their homology, exchanging of one PBP for another in a FRET sensor requires a whole new round of sensor optimization [45]. Similarly, FPs typically cannot be easily exchanged without perturbing the sensor's performance [42,59]. Our group recently introduced a novel strategy for engineering FRET sensors that uses self-associating variants of fluorescent domains [80]. In this approach, a ligand-induced protein-protein interaction is mutually exclusive with an interaction between the two fluorescent proteins. Mutations that allow the intramolecular interaction of the fluorescent domains were first discovered in a FACS-based screening of a CFP/YFP-containing caspase sensor by Daugherty and colleagues [41]. The original aim of that study was to discover mutations that improved the intrinsic fluorescent properties of the fluorescent domains for FRET [41]. The authors discovered a FRET pair (CyPet-YPet) that yielded a dramatically improved caspase sensor that underwent a 20-fold change in emission ratio upon caspase cleavage, compared to a 3-fold change in an ECFP-EYFP-based sensor. Although both FPs were somewhat brighter than their ECFP and EYFP parent proteins, we and others subsequently showed that most of this enhancement can be attributed to an intramolecular interaction of CyPET with YPet, a conformation that brings the fluorophores as close together as sterically allowed [81,82]. We showed, through a systematic study of a model construct consisting of ECFP and EYFP connected by a long, flexible GGSGGS linker, that it was the mutation S208F on both ECFP and EYFP that was responsible for a hydrophobic interaction between the domains [81]. V224L, another mutation discovered in the original CyPET-YPet FACS-based screening, was found to enhance the FRET efficiency in the dimerized state, likely through a subtle effect on the dimer orientation, but was not capable of mediating the interaction on its own [81]. Crucially, upon enzymatic cleavage of the linker between ECFP and EYFP, the interaction was disrupted (Figure 5A). Thus, the interaction only operated at the mM effective concentration provided by the tethering of ECFP and EYFP to one another. Although the interaction was not directly quantified, the *K*_d_ should be well above the 1 μM protein concentration used in this study [81].

One of the first applications of these “sticky” fluorescent domain interactions was in the improvement of the CALWY FRET sensor for Zn^2+^, previously developed in our group [83]. The sensor carried two domains (WD4 and ATOX1) that together coordinate Zn^2+^, *via* cysteine pairs on both domains. WD4 and ATOX1 were connected by a long, flexible linker and an ECFP was fused to the N-terminus of WD4, while an EYFP was fused to the C-terminus of ATOX1. The emission ratio (EYFP/ECFP) was 21% lower in the presence of Zn^2+^ than in its absence [83]. Introduction of the hydrophobic mutations S208F/V224L resulted in a 6-fold improvement of the DR. This improvement is due to the increase in FRET in the Zn^2+^-free state, where the hydrophobic FP mutations bring the FPs in close proximity (Figure 5B). Upon addition of Zn^2+^, the FP-FP interaction in the eCALWY sensor is disrupted as this state is mutually exclusive with the state in which Zn^2+^ is coordinated between the metal binding domains (Figure 5B). Interestingly, the S208F/V224L mutations resulted in a 10-fold attenuation of the sensor's affinity for Zn^2+^, an observation that is best explained by a thermodynamic coupling between the two mutually exclusive states. As it was found that the resulting 2 pM affinity for Zn^2+^ was still too high, a combined strategy of ATOX1-WD4 linker truncations [83] together with a cysteine to serine mutation of WD4, allowed the generation of series of six sensor variants with attenuated affinities ranging from 2 pM to 3 nM [84]. The sensors helped to accurately determine the free Zn^2+^ concentration in the mammalian cytosol (∼400 pM) for the first time [84].

In a second example, the sticky mutations were applied to the design of a FRET sensor based on the LBD of FXR, the nuclear receptor for bile acids. Like the NR-based FRET sensors discussed above (Figure 4C), the bile acid sensor (BAS) exploited a ligand-induced LBD-cofactor peptide interaction. In this case this interaction served to disrupt the “sticky” FP intramolecular interaction, creating a molecular switch triggered by bile acid binding [85]. In the bile acid FRET sensor, the LBD was tightly fused to Cerulean at the LBD's N-terminus, while the LBD's C-terminus was connected via a long flexible linker to Citrine. Finally, a co-activator peptide, containing the LXXLL motif, was fused to Citrine's C-terminus (Figure 5C). In the absence of bile acid, the Q204F mutations (equivalent to S208F) on Cerulean and Citrine ensured a high level of FRET due to the intramolecular complex formation of these fluorescent domains. Addition of bile acids resulted in a large reduction of FRET (DR = 100%), due to the induced LBD-co-activator interaction, which was sterically incompatible with the fluorescent domain complex [85].

The “sticky” FPs have also been employed in FRET sensors for antibody detection [86]. In these sensors, two peptide epitopes are introduced at the ends of a long, semi-flexible linker between Cerulean and Citrine fluorescent domains bearing the Q204F mutation (Figure 5D). The epitope binding sites of an antibody are separated by ∼100 Å so that binding of a single antibody to both epitopes results in disruption of the FP-FP complex. An initial design, using a fully flexible linker consisting of 9 repeats of GGSGGS, was not functional. This is probably because bridging the 100 Å distance required a full extension of the linker and conformations bringing the linker in the fully extended state occupy just a small proportion of the entire distribution of possible linker conformations. Insertion of two α-helical blocks within the linker solved the problem and a sensor was produced that displayed a 5-fold reduction in its Citrine/Cerulean emission ratio [86].

Koide and colleagues established a novel protein engineering technique, affinity clamping, whereby a Fibronectin type III domain is evolved by phage display to form a complex with a peptide sequence of choice and a natural peptide binding domain (PDZ) [87]. These domains, which are connected by a short linker, could be converted to FRET sensors of specific peptides, by fusing CyPet and Ypet to either end of the construct. In absence of peptide, CyPet and YPet were intramolecularly dimerized, resulting in a high level of FRET. Addition of a specific peptide resulted in complex formation between Fibronectin type III, peptide and PDZ, resulting in disruption of CyPet-YPet complex [88] with a DR of 130%.

More recently, the “sticky” FP approach has been expanded to include red fluorescent proteins (RFPs) [89]. Most FRET sensors developed thus far employ CFP and YFP, preventing multiparameter imaging by simultaneous use of multiple FRET sensors at the same subcellular location [90]. One solution has been to develop new, spectrally distinguishable orthogonal FRET pairs from scratch [91,92]. Though feasible, this approach does not take advantage of the many optimized CFP-YFP-based sensors that have already been developed, which are preferably used in combination with a fully red-shifted sensor. However, attempts at employing RFPs such as mOrange and mCherry resulted in sensors with modest [93–95] or no [42] ratiometric response. Our group recently discovered mutations that introduced a hydrophobic interaction between mOrange and mCherry. The presence of R125I on both red fluorescent domains mediated a weak hydrophobic interaction that was similar to the interaction mediated by S208F in CFP and YFP: exchange of the sticky CFP/YFP FRET pair with the sticky mOrange/mCherry FRET pair resulted in functional sensors for protease activity, Zn^2+^ [89] and bile acids (unpublished results). The approach allowed co-imaging of two FRET sensors, differing from one another by orders of magnitude in affinity for Zn^2+^, in the same cellular compartment. Promisingly, the protein engineering strategy appeared to be generically applicable to other members of the mFruit family of RFPs; a protease sensor consisting of ‘sticky’ variants of LSSmOrange [96] and mCherry also displayed a robust ratiometric response [89].

It is important to realize that many of the classically designed FRET sensors, such as Cameleon and its derivatives have been carefully optimized to exploit subtle changes in the conformation of the recognition domain, so that introduction of dimerizing mutations is unlikely to improve them. Kotera *et al.* reported that introduction of S208F and V224L into the fluorescent domains of Ca^2+^ sensors YC3.60 or TN-XL reduced these FRET sensors' DR more than 5-fold [62]. GFP derivatives have a known tendency to dimerize, with a ∼100 μM *K*_d_ [97]. Interestingly, the same sensors required a subtle level of interaction between the fluorescent domains in the Ca^2+^-bound state of the sensor, as introduction of monomerizing mutation A206K [97] also attenuated the DR of YC3.60 and TN-XL [62]. The KCP-1 and KCP-2 FRET sensors of PKC kinase activity are another example of sensors that require FPs' weak dimerization tendency for their mechanism of action. Introduction of A206K into these sensors' fluorescent domains completely attenuated the sensors' ratiometric response. A control experiment ruled out intermolecular FRET, indicating intramolecular dimerization was the dominant factor [42].

**Figure 6.**
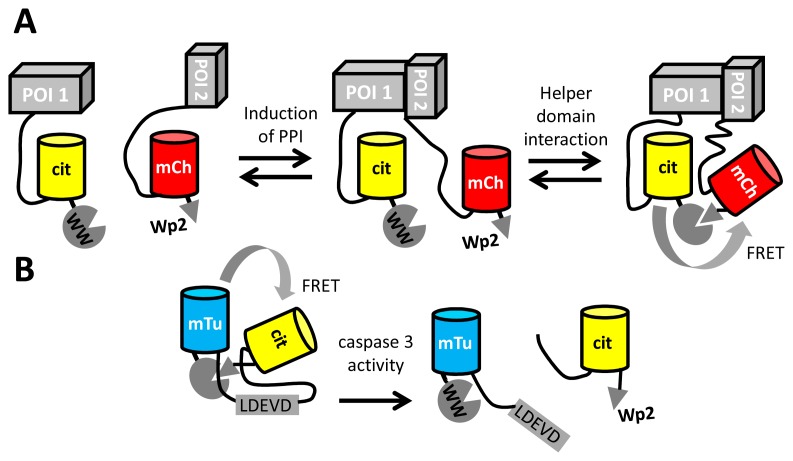
“Weak helper interaction” strategy for the generation of efficient FRET probes. (**A**) The FRET-based detection of a protein-protein interaction (PPI) between one protein of interest (POI1) and a second POI (POI2) is improved through the use of helper domains that bring the FP domains in closer proximity. Induction of a PPI leads to efficient FRET from mCitrine (cit) to mCherry (mCh). The helper interaction only occurs at high effective concentration resulting from the PPI of the POIs. (**B**) The helper interactions also improved the dynamic range of intracellular caspase sensors that use mTurquoise2 (mTu) and Citrine (cit) [98].

Apart from the detection of small molecules and enzyme activity, FRET between fluorescent proteins may also be used to detect protein-protein interactions (PPI), by tagging one interactant with a FRET donor and the other with an acceptor FP. To achieve sufficient FRET in the PPI state, it is necessary to tightly couple the fluorescent tag to the protein of interest, yet this may cause steric hindrance of the PPI. Longer linkers, while minimizing interference of the PPI, result in only a very weak FRET signal due to the distance dependence of FRET. Recently, Serrano and colleagues provided a solution to this problem, by using a peptide-domain interaction as a secondary interaction for the fluorescent domains [98]. The peptide (Wp2) was fused adjacent to one FP, while a small domain (WW) with affinity for the peptide was fused adjacent to the other fluorescent domain (Figure 6A). The peptide-domain interaction (from a set of previously characterized pairs) was chosen so that the interaction occurred only in the presence of a primary PPI. This “helper” interaction, with a *K*_d_ of around 170 μM, improved the detection of PPIs by fluorescence lifetime imaging, by increasing FRET efficiencies about two-fold, from ∼20% to ∼40% [98]. The same helper domains were also used to improve the dynamic range of a caspase FRET sensor consisting of mTurquoise2 and mCitrine. (Figure 6B).

## 5. Development of FRET Sensors Using High Throughput Screening

The improvement of FRET sensors by repeated expression, purification and analysis of new sensor variants can be laborious and time-consuming. A more efficient method may be to simultaneously screen a large number of variants for ratiometric response to ligand addition. An early example of the use of high throughput screening for improving such sensors was provided by Hires *et al.* [99]. GluSnFr is a sensor for the neurotransmitter glutamate and employs the periplasmic binding protein (PBP) GltI, but displayed a poor DR of less than 10% [99]. GluSnFr was improved through screening of 175 linker truncation variants, resulting in SuperGluSnfr, with a 46% DR [99]. The poorly responsive EKAR FRET sensor for ERK kinase activity [73], also improved upon by Komatsu *et al.* [74] (see Section 3), was also subjected to a systematic screening. The effects of different combinations of donor FP (wt mTFP1 or four different cp variants thereof) and acceptor FP (wt Venus or different cp variants thereof), were tested in the context of four different topologies of the two FPs, substrate peptide and phosphobinding domain [75]. A total of 100 different variants were screened directly in mammalian cells using a fluorescence plate reader. Notably, Pertz and coworkers discovered a variant displaying a 2-fold improvement in DR over the original EKAR sensor [75].

With the goal of establishing a rapidly implementable, universal strategy for FRET sensor optimization, Schulz and colleagues created a set of 36 vectors carrying FRET sensor backbones with different combinations of donor (ECFP, mTurquoise and mTurquoise carrying a C-terminal deletion) and acceptor (Venus and four different cpVenus variants) fluorescent domains, together with different linker lengths, between which a kinase of interest was to be cloned [100]. Particularly innovative was the use of reverse transfection, where DNA is spotted onto glass microslides, before transfection is achieved by culturing mammalian cells over this DNA. The authors generated FRET sensors for the activities of two different calcium/calmodulin associated kinases. The DR of sensors based on death-associated protein kinase 1 (DAPK1), varied from 10% for the worst variant to 55% for the best variant, proving the value of this systematic screening strategy [100].

Library sizes in FRET sensor development are relatively small when compared to the sizes typically encountered in the development of fluorescent proteins. The screening of sensors is a more complex operation than that of FPs, as sensors need to be screened under two different conditions, in the on and in the off state. In screening for FRET sensors of enzyme activity, one can exploit the fact that sensor variants can be imaged both before and after induction of the enzyme-of-interest. Campbell and colleagues discovered improved FRET sensors for the activity of histone methylating enzymes by screening bacterial colonies under two expression regimes: expression of a sensor variant alone or in presence of a co-expressed methylating enzyme [101]. Two libraries with variation in the linker sequences were screened in succession, the first with 392 possible members, the second with 640 possible variants. The throughput was limited due to the need to manually spot each colony on a co-expression and a repression plate. Only 270 colonies for the first library and 540 colonies for the second library were actually screened, suggesting that many potentially useful variants were missed, especially since some variants would have occurred more than once in the screening. Perhaps as a result of that, improvement in DR was 2.3-fold [101].

The same group also reported the use of high throughput, bacterial colony-based screening to improve FRET sensors for kinase activity [102]. Earlier work on a FRET sensor for protein kinase B/Akt (PKB) named BKAR [103] had failed to improve the sensor's small DR. A library of ∼400 BKAR variants was created, in which the linker sequence between the peptide substrate and the phosphobinding domain was varied, and different CFP and YFP variants were tested. Dual expression plasmids were used in which BKAR was under an IPTG-inducible promoter, while a constitutively active kinase was under control of an arabinose inducible promoter. Bacterial colonies, grown on an IPTG-containing medium, were first imaged in absence of kinase expression. Subsequently, expression of kinase within the bacterial cells was induced by spraying arabinose on the colonies. After 2 h, during which time kinase formed and was allowed to phosphorylate the BKAR variants, the colonies were imaged again. The best BKAR variant obtained had a DR that represented a disappointing 1.3-fold improvement over the original sensor. Unfazed, the authors attempted to optimize a second kinase sensor, one that reported the activity of CDK1 in complex with cyclin B1. Using the same approach, the authors discovered a highly responsive variant with a DR of 70%, a 4.5-fold improvement over the original sensor [102].

**Figure 7.**
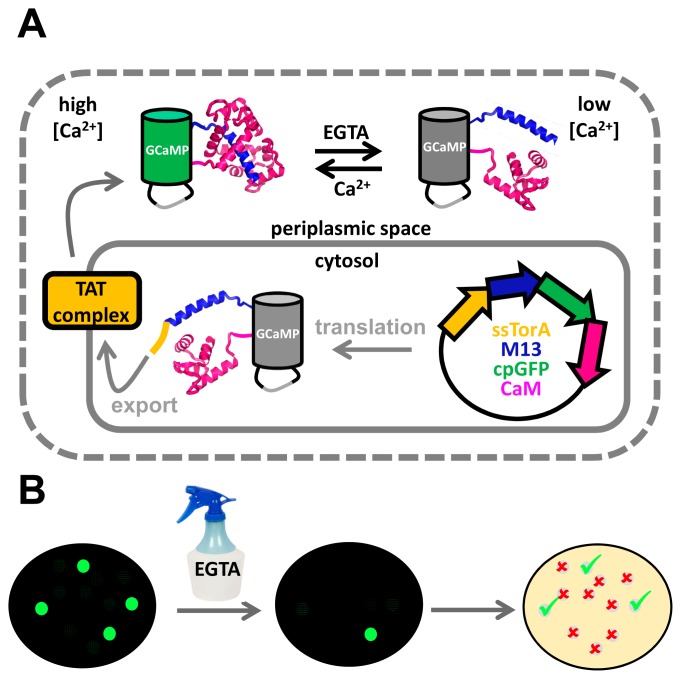
Development of GECO-type Ca^2+^ fluorescent sensor proteins. (**A**) Fusion of an N-terminal Tat signal sequence targets GCaMP3 to the periplasm, where its fluorescence can be modulated by changing the external [Ca^2+^] through the addition of EGTA. (**B**) *E. coli* colonies carrying GCaMP3 variants are screened for EGTA-induced loss of fluorescence. Colonies showing bright fluorescence in the absence and dim fluorescence in the presence of EGTA are selected for further analysis or subsequent rounds of evolution. Figure adapted from [104].

For the high throughput screening of sensors of small molecules, sensors must somehow be exposed to high and low concentrations of ligand. Manipulating the ligand concentration in the cytosol, where sensor variants would normally be located, can be challenging due to the impermeable cytoplasmic membrane. Campbell and co-workers, seeking to improve single FP-based Ca^2+^ sensors, realized that the cytosolic concentration of Ca^2+^ in *E. coli* is low and cannot easily be manipulated, but that the bacterial outer membrane is permeable to small molecules and metal ions, rendering the periplasmic space far more accessible to external manipulation [22]. Using the twin arginine translocation pathway, a system that transports fully folded proteins across the cytoplasmic membrane, single FP-based Ca^2+^ sensors were brought into the periplasmic space (Figure 7A). Once there, the Ca^2+^ concentration could be manipulated by spraying a fine mist of EGTA solution over the bacterial colonies growing on an agar plate (Figure 7B). Impressively, the authors managed to screen up to 200,000 colonies. Crucially, this strategy allowed manipulation of the sensors' environmental conditions, while keeping the protein associated with its encoding DNA for later analysis. Not only was the DR of the best performing indicator at that time (GCaMP3) improved 2-fold, the authors also introduced novel red-shifted and blue-shifted variants, as well as an excitation-ratiometric variant displaying a 110-fold change in excitation ratio [22].

Recently, Thrustrup *et al.* presented an impressive strategy by which bright Ca^2+^ FRET sensors (called Twitch) were produced with a DR of up to 1000% [105]. The initial design, Twitch 1, consisted of a fusion of ECFP, a minimal Ca^2+^-binding domain (toadfish troponin C) and EYFP and yielded, after selection from a 49-member linker library, a sensor with a DR of 400%. Next the authors devised a clever way to rapidly screen additional variants. Colonies were blotted onto filters, before the filters were sprayed with ionomycin and polylysine, permeabilizing the cells. Using automated image analysis that could recognize the discrete colonies, emission ratios were then detected before and after application of 100 mM CaCl_2_. In total, two libraries of almost 100,000 colonies each were screened, the first for optimal linkers, the second for optimal mutations at hotspots of the troponin C domain, selected on the basis of an NMR solution structure. Finally, the ECFP donor was replaced with the much brighter and photostable mCerulean3 [106] or mTurquoise2 [107]. Although this initially led to a large reduction in DR, additional screening with extended linkers recovered the sensors' responsiveness.

Genetically encoded sensors sometimes yield promising results *in vitro* or in mammalian cell cultures yet then fail in more demanding settings such as neurons or mice. Several groups have demonstrated the importance of carrying out systematic screening of FRET sensors *in situ* in the cell lines in which the sensor is to be used. For example, it was found that there was a poor correlation between Ca^2+^ sensor GCaMP's DR measured in bacterial lysate and the DR measured in intact HEK293 cells [12]. Many of the existing Ca^2+^ sensors are not capable of responding to the rapid neuronal Ca^2+^ switching kinetics. Screening 447 variants in rat hippocampal neurons revealed GCaMP variants with unprecedented sensitivity and fast dynamics [13]. GCaMP6f had a two-fold faster rise time and a 1.7-fold faster decay time than the previous generation GCaMP5G [13]. Similarly, the Twitch series of Ca^2+^-FRET sensors was also improved through the screening of 120 variants in rat neurons that were exposed to electrical field stimulation [105].

## 6. Conclusions and Outlook

Genetically encoded FRET sensors for intracellular ligands offer a number of benefits including modular sensor design, a sensor concentration-independent output signal and accurate subcellular targeting. In addition, genetic encoding allows convenient distribution among cell biologists, as an increasing number of FRET sensors have become available through depositories such as AddGene. The development of responsive FRET sensors has proven to be challenging in many cases but in recent years has benefitted both from rational design strategies and from directed evolution-like approaches. Currently, FRET sensor measurements are mainly limited to cell culture models, leaving the physiological context of cell signaling largely unexplored. Deep tissue *in vivo* imaging will require development of bright, red-shifted fluorescent proteins, detectable through the optical window found between 600 and 1200 nm. However, RFPs with emission beyond 600 nm remain relatively dim [108–111] although we expect that brighter, more red-shifted RFPs will be discovered in the coming years. By employing the protein engineering strategies described above, it should be possible to incorporate these into novel FRET sensors, even using non-canonical fluorescent proteins that are structurally unrelated to GFP [110,112].
